# Attention Deployment to the Eye Region of Emotional Faces among Adolescents with and without Social Anxiety Disorder

**DOI:** 10.1007/s10608-020-10169-2

**Published:** 2020-10-23

**Authors:** Nicole N. Capriola-Hall, Thomas H. Ollendick, Susan W. White

**Affiliations:** 1 Center for Youth Development and Intervention, University of Alabama; Tuscaloosa, AL 35487; 2 Virginia Tech; Child Study Center, Department of Psychology, Blacksburg, VA 24061

**Keywords:** Social anxiety disorder, Adolescents, Eye-tracking, Attention, Emotion

## Abstract

**Background:**

Avoidance of the eye region, especially of faces showing anger, may maintain social anxiety symptoms by negatively reinforcing expectations and fears associated with social situations. Eye-tracking research, however, has yet to explicitly examine differences in attention allocation to the eye region of emotional faces among adolescents with social anxiety disorder (SAD).

**Methods:**

Gaze patterns were explored in a sample of youth with and without SAD matched on age and sex.

**Results:**

Adolescents with SAD were quicker to fixate, and maintained their initial gaze longer, to the eye region, regardless of emotion, relative to teens without SAD. Group-level differences also emerged for initial fixation duration directed to the eye region of angry faces (when compared with happy faces).

**Conclusions:**

These findings suggest that vigilance to the eye region of faces, especially angry faces, (when compared with happy faces) is characteristic of adolescents with SAD. Adolescents with SAD seem drawn to the eye region, more so than teens without SAD.

Cognitive models of social anxiety suggest that symptoms are maintained by deficits in social information processing (Rapee & Heimberg, 1997) and that social anxiety disorder (SAD) is characterized by abnormalities in visual processing of threat related stimuli (Armstong & Olajunti, 2012; Schmidtendorf et al., 2018). Specifically, eye tracking studies using emotional face pairs have found that compared with healthy controls, children and adolescents with SAD demonstrate an initial orienting bias towards angry faces relative to neutral faces (Capriola-Hall et al., 2019; Seefeldt et al., 2014). However, findings by Schmidtendorf and colleagues (2018) did not find evidence for hypervigilance using free-viewing eye-tracking paradigms. These authors found that children (ages 9–13, *M* age = 11.4) with SAD initially fixated their gaze less often on angry faces relative to happy and neutral faces; however, the directionality of attention changed following schema activation (i.e., participants told they were going to give a speech task which prompted fears of social evaluation). Overall, these findings provide initial evidence for patterns of attention allocation associated with SAD in children and adolescents and suggest the importance of considering the temporal course of attention bias across stimulus presentation (i.e., both initial fixation and over the time course of stimulus exposure; Schmidtendorf et al., 2018).

Although much research has examined attention bias to threatening faces in children and adolescents with anxiety diagnoses (Capriola-Hall et al., 2019; Schmidtendorf et al., 2018; Seefeldt et al., 2014; Shechner et al., 2013), little is known about attention to the eye region *specifically* among adolescents with SAD. This is particularly surprising since adults with SAD tend to avoid direct eye contact during social interactions (Schneier et al., 2011). Indeed, during social interaction, a considerable amount of time is spent fixating on the eyes both with healthy individuals as well as those with anxiety more broadly, including SAD (Grossmann, 2017; Haxby et al., 2002; Michalska et al., 2017). Eye gaze is posited to serve as an important social cue (Frischen et al., 2007) and serves several critical functions in regard to social processing (i.e., provision of information related to a person’s mental state and the facilitation of communication and regulation of the flow of conversation; see Pitskel et al., 2011). More specifically, the eye region is thought to contain more social information than any other area of the face (Ristic et al., 2005), suggesting that visual preference towards the eyes is a facilitator of social interaction and signals preparedness for social interaction. This is especially salient in social situations in which the face conveys threat (Green et al., 2003; Öhman et al., 2001). Extant research also suggests that the eye region of angry faces signals social disapproval and social evaluation (Öhman, 1986). Thus, the avoidance of eye contact in social anxiety might serve as an attempt to evade social threat and to regulate excessive fear (Schulze et al., 2013).

To date, only a handful of studies have used eye-tracking methodology to specifically measure visual attention to the eye region among generally anxious and socially anxious samples. In one study, severity of general anxiety symptoms in a sample of children was related to avoidance of the eye region (Michalska et al., 2017). In an adult sample, Moukheiber and colleagues (2010) showed that avoidance of eye contact is a robust phenotypic marker of SAD. A recent study by Keil and colleagues (2018) in a sample of children with SAD demonstrated that social anxiety severity was negatively associated with duration of first fixations (i.e., shorter first fixation duration). Although group-level differences in visual attention to the eye region emerged for children with SAD versus controls, these findings were not limited to angry stimuli (Keil et al., 2018). These results suggest that the eye region is a salient facial feature and perhaps threatening, given evidence for visual avoidance among children with SAD (Keil et al., 2018). Overall, these studies provide evidence for a relationship between anxiety and avoidance of the eye region. However, no research to date has focused specifically on visual attention to the eye region among adolescents with SAD versus those without SAD. Our sample composition is important given social anxiety symptoms – including concerns about peer evaluations increase during the adolescence, with the peak onset of SAD being around 13 years of age (Beesdo-Baum & Knappe, 2012).

We sought to examine the relationship between visual attention to the eye region of both emotional (angry and happy) and neutral faces among adolescents with SAD and those without SAD. Consistent with past research suggesting avoidance of the eye region (Keil et al., 2018; Michalska et al., 2017), we hypothesized that avoidance of the eye region would be unique to the SAD group. Although there is evidence for vigilance towards threat being observed in youth with SAD (Capriola-Hall et al., 2019), these studies have not focused on the eye region specifically. We also predicted that avoidance of the eye region would be more pronounced for angry faces compared to neutral or happy faces. The inclusion of happy and neutral faces is consistent with past-eye tracking studies by Seefeldt and colleagues (2014) and Schmidtendorf and colleagues (2018) and allows for the examination of whether attention towards the eye region is associated with the emotional valence of the facial stimuli (Keil et al., 2018).

## Method

### Participants

Pre-treatment data from a clinical sample of adolescents with SAD (*n* = 28, *M* age = 14.07) were drawn from a randomized controlled trial of a computerized treatment to reduce social anxiety symptoms (Ollendick et al., 2019). A separate non-SAD group (*n* = 25, *M* age = 13.56) was also recruited, specifically for this study. All participants (*n* = 53; *M* age =13.83) were between the ages of 12–16 and free of co-occurring intellectual disability. Participant characteristics are presented in Table 1.

### Procedure

Study procedures and protocols were approved by the university’s institutional review board for human subject research. All parents provided informed consent and youth gave assent prior to beginning the study. Adolescents and their families were recruited through various sources including the institution’s psychology department’s child participant database, local child psychiatric and mental health clinics, school health services, primary care practices, and other online and print advertisements. Participants received a small honorarium for their time investment.

#### SAD Group.

Adolescents with SAD were recruited as part of a NIMH-funded treatment study (Ollendick et al., 2019). Upon initial contact, parents of potential study participants completed a brief telephone screen in order to determine study eligibility. Enrolled families participated in a pre-treatment assessment session. Inclusion criteria for participation consisted of: 1) diagnosis of SAD as determined by a semi-structured diagnostic interview (Anxiety Disorders Interview Schedule for DSM-IV-Child and Parent Versions (ADIS-IV-C/P; Silverman & Albano, 1996); 2) full scale IQ of 80 or above, as determined by the Wechsler Abbreviated Scale of Intelligence, 2nd edition (WASI-2; Wechsler, 2011); 3) stable psychotropic medication as determined by no dosage changes for at least four weeks (*n* = 1, prescribed anti-depressant); and 4) no ongoing intervention for social anxiety related concerns. Participants were excluded if they met criteria for autism spectrum disorder, childhood schizophrenia, and/or problems that warranted more immediate care (e.g., suicidal ideation). As part of the assessment, adolescents completed an eye-tracking task and a battery of questionnaires including the Screen for Child Anxiety Related Disorders, Child Version – SCARED (Birmaher et al., 1997) and the Brief Fear of Negative Evaluation Questionnaire (BFNE; Leary, 1983). Only pre-treatment assessment data were analyzed in this study.

#### Non-SAD Group.

Upon contact, potential participants’ parents completed a brief phone screen in order to determine initial eligibility. In addition to being between 12 and 16 years of age, inclusion criteria for the non-SAD group were: 1) absence of psychiatric problems as determined by parent report on the initial telephone screener, and 2) absence of elevated SAD symptoms as assessed by a total anxiety score < 25 and < 8 on the social anxiety subscale of the SCARED (Birmaher et al., 1997). Participants without SAD completed a subset of the same questionnaires as the SAD group (overlapping measures described below) and the same eye-tracking task as the SAD group.

### Measures

#### Screen for Child Anxiety Related Disorders, Child Version (SCARED; Birmaher et al., 1997).

The SCARED examines a broad range of anxiety symptoms and is comprised of 41 items rated on a 3-point Likert scale. The SCARED yields scores for Panic Disorder, Generalized Anxiety, Separation Anxiety, Social Anxiety, and School Avoidance subscales, in addition to a Total Score. The test-retest reliability, internal consistency, and discriminant validity are well-documented (Birmaher et al., 1997). In the current sample, internal consistency was within acceptable ranges for both the SAD group (α = 0.88) and the non-SAD group (α = 0.77) for the SAD subscale. For the total score, internal consistencies were within the good to excellent range (α = 0.93) for SAD group and (α = 0.86) for the non-SAD group.

#### Brief Fear of Negative Evaluation Questionnaire (BFNE; Leary, 1983).

The BFNE is an abridged version of the full FNE (Watson & Friend, 1969), consisitng of twelve items that assess worry or fear about negative evaluation from others. BFNE items are coded on a Likert scale ranging from 1 (not at all characteristic of me) to 5 (extremely characteristic of me). The BFNE correlates highly (.96) with the original FNE and has excellent internal consistency (alpha = .90; Leary, 1983). Carleton and colleagues (2011) found that using only the eight items that have straightforward wording (i.e., not the four reverse-scored items) results in the best diagnostic sensitivity and reliability. As such, only the eight straightforward worded items were summed for the present study. Past studies have supported the use of the BFNE in examining FNE within adolescent samples (Capriola, Maddox, & White, 2016; de Hullu et al., 2011). In the current sample, internal consistency was excellent for both the SAD group (α = 0.95) and the non-SAD group (α = 0.92) for the BFNE total score.

### Eye-Tracking Apparatus, Stimuli, and Data Processing

**Figure 1.**
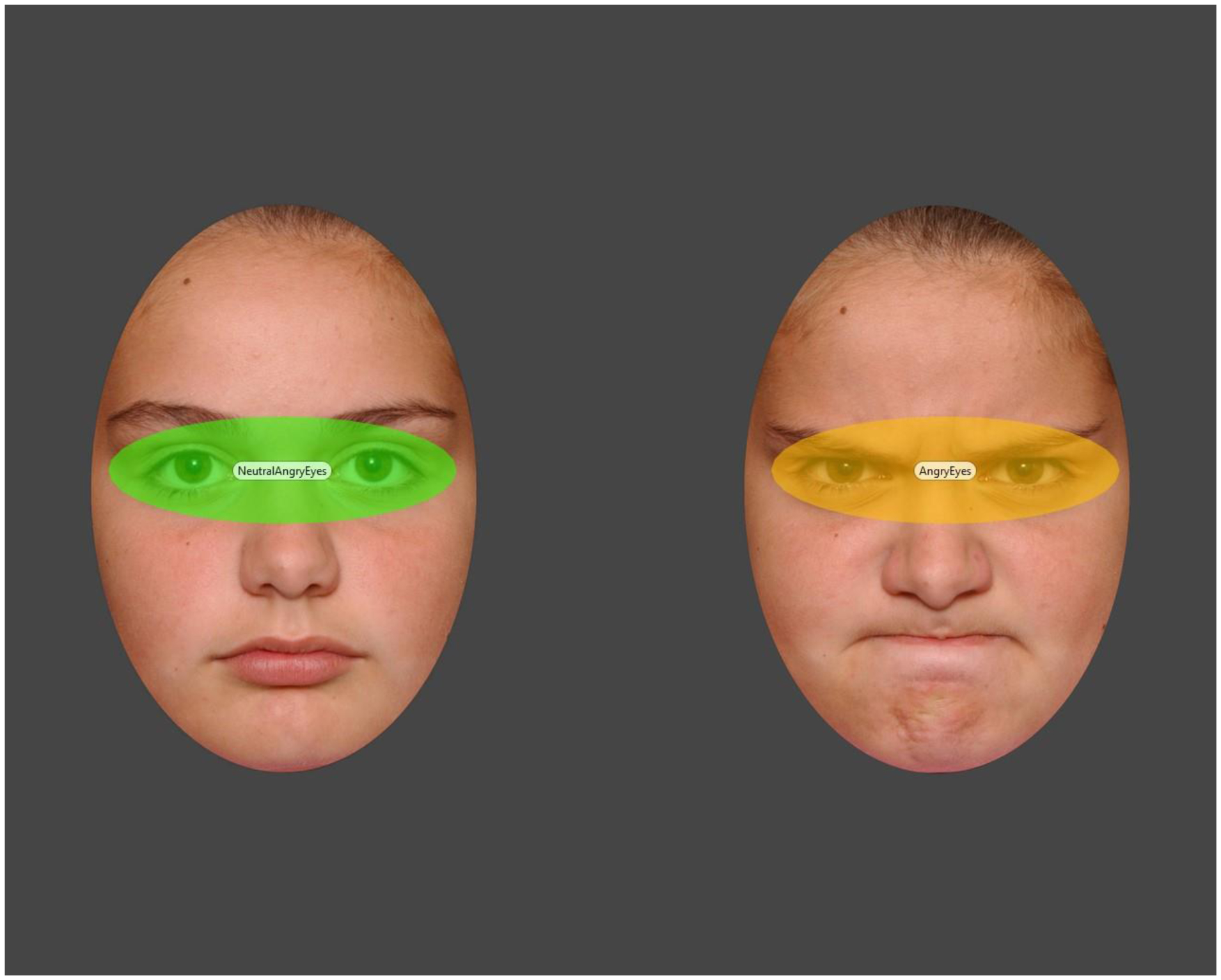
AOI for the Eye Regions. Note. Green and yellow ovals represent the created areas of interest for the eye region.

Eye-tracking was completed using a Tobii T60 XL eye-tracker in order to track eye movement and foveal fixation. A standard calibration procedure was completed at the beginning of eye-tracking data collection. The eye-tracker’s calibration system was set to 0.5 degrees of accuracy with less than 0.3 degrees of visual drift. The five-point calibration procedure involved tracking a moving red circle located at five predefined locations. After the five circles appeared, the Tobii eye-tracker provided a pictorial representation of calibration quality, with small dots in the center of each circle representing high quality, and missing dots and/or lines extending from one or more dots representing lower quality. The examiner visually inspected each display before advancing the participant to the eye-tracking task. Any missing calibration points, or points with excessive error, were recalibrated to achieve acceptable quality. Following calibration, participants were prompted to freely look at the stimuli (i.e., passive viewing) while keeping their head still. This paradigm allows for the examination of multiple attention processes [e.g., hypervigilance to threat, initial maintenance of attention to threat, and dwell time across stimulus presentation (Armstrong & Olatunji, 2012; Dodd et al., 2015)]. The Tobii eye tracker collected the raw eye movement data points, which were processed into fixations. A fixation was defined as a set of consecutive gaze coordinates for at least 100 milliseconds. The areas of interest (AOIs) were predefined, using the oval-shaped AOI tool available in the Tobii T60 (Studio Professional) platform, for each face by the first author (Figure 1). Given the aims of the current study, we focused solely on visual attention directed towards the eye region. Although not related to the study’s primary aims, we examined whether greater visual attention (i.e., greater fixation direction percentages towards and greater fixation duration) would be allocated to the eye region relative to the rest of the face or the mouth region^1^. This allowed us to determine whether effects observed in the current study were unique for gaze to the eye region or simply representative of patterns of attention to the face in general or the mouth region. The duration of fixation, time to first fixation, and first fixation duration to the eye region were calculated using an in-house MATLAB code. Fixation data were excluded if the participant did not fixate on the central fixation cross prior to experimental screen onset. Across all calculated metrics, data were removed if major tracking loss was observed (e.g., greater than 50% of stimulus presentation time; Wieckowski & White, 2017).

The eye-tracking stimuli were from the National Institute of Mental Health (NIMH) Child Emotional Faces Picture Set (NIMH-ChEFS; Egger et al., 2011) consisting of adolescent faces. All faces demonstrated at least 70% rater agreement of presented emotions (Coffman et al., 2015). Each face was presented in an equally sized oval shape (each face 19.05 cm long X 16.51 cm wide, with 11.43 cm of grey space between the two faces, all subtending 37° visual angle) against a grey background. Each trial contained a pair of photographs of the same actor, with one photo depicting an emotional face (i.e., angry or happy) and the other depicting a neutral facial expression. This methodology is consistent with previous eye-tracking research (e.g., Shechner et al., 2013; Wieser et al., 2009) which posits that attentional biases are more likely to occur when more than one stimulus is competing for attention (In-Albon et al., 2010). A centered X was presented for 1 second, immediately followed by a face-pair shown for 3 seconds. After the face-pair, a grey screen was presented for half a second. Within the free viewing task, 32 face pairs (i.e., 16 anger-neutral and 16 happy-neutral) were presented in a counterbalanced fashion, and the emotional face appeared equally on both sides of the screen. Equal numbers of male and female faces were presented (i.e., 8 unique face-pairs).

In order to comprehensively measure both potential biases in initial orientation and sustained attention to social stimuli, we calculated multiple indices of attentional bias toward the eye region (i.e., latency, first fixation direction proportions, duration for first fixation, fixation duration across six time epochs (i.e., each 500ms), and total fixation duration), to examine both the spatial (i.e., eye region) and temporal (i.e., biases in initial orientation versus sustained processing) components of attention allocation (Armstong & Olatunji, 2012). Latency was quantified in the current study as the time from onset of face stimuli until the first fixation to either the emotional or neutral eye region (Shechner et al., 2013). First fixation direction was defined as the proportion of first fixation direction (i.e., number of trials gaze was first directed to angry eye region divided by total number of trials with eye movements to angry-neutral face pairs). This was also calculated separately for the eye region of happy-neutral face pairs. First fixation direction is a metric which measures the tendency to orient attention first to one type of stimuli (see Gamble & Rapee, 2010; Shechner et al., 2013). Duration for first fixation, which has been used in past eye-tracking studies to assess disengagement and/or maintenance of attention (Buckner, Maner, & Schmidt, 2010; Dodd et al., 2015), was also calculated. Fixation duration was defined as the total length of time (in ms) that the participant fixated on the eye region of the stimulus based on the average of both eyes. Total fixation duration is regularly used as a measure of preference for looking at one stimulus over another (Buckner et al., 2010; White et al., 2015). Greater fixation duration toward socially threatening stimuli was viewed as a measure of sustained visual attention towards threat (i.e., vigilance) whereas avoidant visual attention is characterized by shorter total fixation duration. This metric allows for an examination of biases over longer periods of stimulus exposure. To assess the temporal dynamics of dwell time, we also explored changes in participant visual attention across six time epochs. Bonferroni post-hoc tests were calculated to investigate significant interactions and main effects for the time interval analyses.

### Data Analysis

Analyses were conducted with IBM SPSS Statistics Version 24.0. Descriptive statistics are presented in Table 1. Consistent with Price and colleagues (2015), a Winsorizing procedure was used to eliminate extreme values while minimizing missing data, an approach that is robust to violations of standard statistical test assumptions (Erceg-Hurn & Mirosevich, 2008) and corrects for undue influence of outlier data points. Values outside 1.5 inter-quartile ranges from the 25th or 75th percentiles (the “Tukey Hinges”) of a given distribution of values across all individuals were rescaled to the last valid value within that range (see Price et al., 2015). Reliability of the eye-tracking measures were calculated using Cronbach’s alpha, consistent with recommendations by Price et al. 2015 (Table 2). Repeated measures analyses of variance (ANOVAs) were undertaken for each of the identified eye-tracking metrics: Latency of first fixation, mean initial fixation duration, mean duration of fixations across stimulus presentation. Specifically, repeated measures ANOVAs with stimulus type (angry versus neutral, happy versus neutral) x group (adolescents with SAD vs. adolescents without SAD) were computed. Independent samples t-tests were conducted to determine whether there were differences in the direction of initial fixation between youth with SAD versus youth without SAD. For mean fixation duration across the six time epochs, a 3-way repeated measures ANOVA with time (six intervals) x stimulus type (angry versus neutral, happy versus neutral) x group was conducted. Analyses were conducted separately for angry-neutral and happy-neutral face pairs given the paired nature of the task (i.e., emotional face always paired with neutral face). However, secondary analyses explored potential differences in emotion specificity on visual attention towards the eye region using a multivariate, repeated measures ANOVA.

## Results

Group differences in participant age, race, and sex were statistically non-significant (Table 1). The group level differences for the completed measures (SCARED and BFNE) were in the expected directions (i.e., youth with SAD demonstrated greater SAD symptoms, total anxiety symptoms, and more fear of negative evaluation relative to youth without SAD). As such, primary analyses were conducted without demographic covariates. Following winsorizing of five data values (four non-SAD participants for the latency to fixate on angry eye region and one SAD participant for first fixation duration on angry eye region), skewness and kurtosis for all primary variables were within acceptable ranges, and visual inspection of the data distribution indicated no concerns with non-normality. Descriptive statistics for the eye-tracking metrics are presented in Tables 3 and 4. There were no significant differences in the amount of data used for analyses between groups, *t*(50) = 1.04, *p* = .304, suggesting data loss did not vary based on group. Further, there was not a significant group difference in number of trials during which participants did not fixate on the centered “X” before stimulus onset, *t*(50) = −.98, *p* = .331 (*M* = 1.85 trials removed, *SD* = 3.03, range = 0–16). The mean number of trials (i.e., >50% gaze towards the eye region) were as follows: angry-neutral (*M* = 9.23, range = 0–16) and happy-neutral (*M* = 7.39, range = 0–16). The mean number of trials for each emotion did not significantly differ by group (*p*s = .149-.474). As noted in Table 2, reliability estimates were calculated for each emotion across the angry-neutral and happy-neutral eye regions. Cronbach’s alpha values for first fixation direction (.69-.71) and latency (.56- .80) were above the lower limits of acceptability. Cronbach’s alpha values were within the good range for the first fixation duration (.86-.88) and total dwell time metrics (.89-.93). Fixation duration internal consistencies ranged from .38-.68 when separated by time interval.

### Attention to the Eye Region versus Mouth Region.

There were significant differences in the first fixation percentages directed towards the eye region compared to the mouth region, specifically, *t*(52) = −7.11, *p* < .001. In about 67% of trials gaze was first directed to the eye region whereas only approximately 18% of trials had first gaze directed to the mouth. Therefore, we find evidence for greater fixation direction proportions towards the eye region relative to the mouth region. This finding didn’t differ by group, *p*s = .175-.432.

A 2x (eye region and mouth region) x2 (SAD vs. non-SAD) x2 (angry, happy) mixed ANOVA with fixation duration as the dependent variable was computed. There was a significant interaction between emotion and AOI (i.e., eye region versus mouth region), *F*(1,192) = 19.28, *p* <.001, partial eta squared = .091. Total fixation duration to the eye region of angry faces (*M* = 644.55ms) differed significantly from fixation total duration to the mouth region of angry faces (*M* = 215.53ms), *t*(98) = 8.66, *p* < .001. However, total fixation duration to the eye region of happy faces (*M* = 506.94ms) didn’t differ significantly from fixation total duration to the mouth region of happy faces (*M* = 438.49ms), *t*(98) = 1.04 *p* = .301. No other significant interactions were observed.

### Latency.

The interaction between emotion and group was not statistically significant, *F*(1,48) = 1.11, *p* = .298, partial eta squared = .023). Results suggested that regardless of group status, the speed at which the adolescents looked towards the eye region varied significantly by emotion, *F*(1,48) = 7.17, *p* =.010, partial eta squared = .130. Adolescents first fixated on the eye region of angry faces more quickly than the paired neutral eye region, *t* = 2.77, *p* = .008, *d* =0.43. The main effect of group was statistically significant, such that adolescents with SAD were quicker to fixate on the eye regions of both angry and neutral relative to the adolescents without SAD, *F*(1,48) = 10.751, *p* =.002, partial eta squared = .183, *t* = 4.82, *p* < .001, *d* =1.32 (Table 3).

For the happy-neutral face pairs, there was no interaction effect and no significant main effect for stimulus type, *F*(1,46) = .97, *p* =.331, partial eta squared = .021. However, the main effect of group was statistically significant, *F*(1,46) = 19.51, *p* <.001, partial eta squared = .298, indicating that adolescents with SAD exhibited a shorter latency than their non-anxious counterparts to happy-neutral eye regions (collapsed across happy-neutral stimulus type).

### First Fixation Direction.

An independent samples t-test revealed non-significant group differences between youth with SAD and youth without SAD in their initial fixations towards the eye region of angry-neutral face pairs, *t*(48) = −.04, *p* = .965, *d* = .015. Similarly, there was no evidence for group differences for initial fixations towards the eye region of happy-neutral face pairs, *t*(49) = −.90, *p* = .375, *d* = .249.

### First Fixation Duration.

As determined by the repeated-measures ANOVA, the interaction between group and emotion (i.e., angry-neutral) was not significant, *F*(1,47) = .05, *p* =.824, partial eta squared = .001. Further, there were not a main effect of emotion, *F*(1,47) = .10, *p* = .753, partial eta squared = .002. The main effect of group, however, was statistically significant, *F*(1,47) = 16.74, *p* = <.001, partial eta squared = .263, indicating that adolescents with SAD sustained their visual attention towards the initially fixated angry and neutral eye regions longer than did those without SAD (Table 3).

The same analysis was conducted for the happy-neutral eye regions. Consistent with results for the angry-neutral eye region, the main effect of group was the only statistically significant effect. Specifically, adolescents with SAD maintained their gaze towards the initially fixated eye region for happy and neutral faces longer relative to adolescents without SAD, *F*(1,47) = 11.21, *p* =.002, partial eta squared = .199.

### Total Fixation Duration.

For fixation duration to angry-neutral face pairs, no group x stimulus type interaction emerged (*p* > .05). The time spent looking towards the eye region for angry-neutral face pairs did not vary significantly by stimulus emotion, *F*(1,48) = .57, *p* =.455, partial eta squared = .012 nor group, *F*(1,48) = .03, *p* =.867, partial eta squared = .001 (Table 3).

For happy-neutral face pairs, average dwell time across stimulus duration differed by stimulus type, *F*(1,48) = 6.03, *p* =.018, partial eta squared = .114. Specifically, adolescents across the groups maintained their gaze significantly longer towards the eye region of neutral faces relative to the eye region of happy faces (*t* =2.46, *p* =. 017, *d* = .292). No main effects for group or stimulus type x group interactions were detected (P > .05).

#### Fixation Duration Across Epochs.

The 3-way time (6 interval) x valence (angry, neutral) x group (SAD, non-SAD) interaction was not significant, *F*(5, 3717.57) = 1.16, *p* = .326, partial eta squared = .010. Only the main effect of time, *F*(5, 3371.31) = 10.16, *p* <.001, partial eta squared = .081, was significant. Bonferroni post-hoc tests determined that only the first epoch differed significantly from all other time intervals (i.e., fixation duration increased following first epoch) given the adolescent is likely spending more time becoming acquainted to the stimuli and thus demonstrating more frequent saccades (i.e., less dwell time).

The same analysis was conducted for the eye region of happy-neutral face pairs. The 3-way time (6 interval) x valence (happy, neutral) x group (SAD, non-SAD) interaction was not significant, *F*(5, 3026.26) = 2.01, *p* = .076, partial eta squared = .017. Only the main effect of time, *F*(5, 3371.31) = 9.05, *p* <.001, partial eta squared = .073, was significant. Specifically, dwell time to the eye region increased over time across both groups, across the stimuli. Similar to the angry-neutral stimuli, only the first epoch differed significantly from the other time intervals.

### Emotion Specificity Analyses.

**Figure 2.**
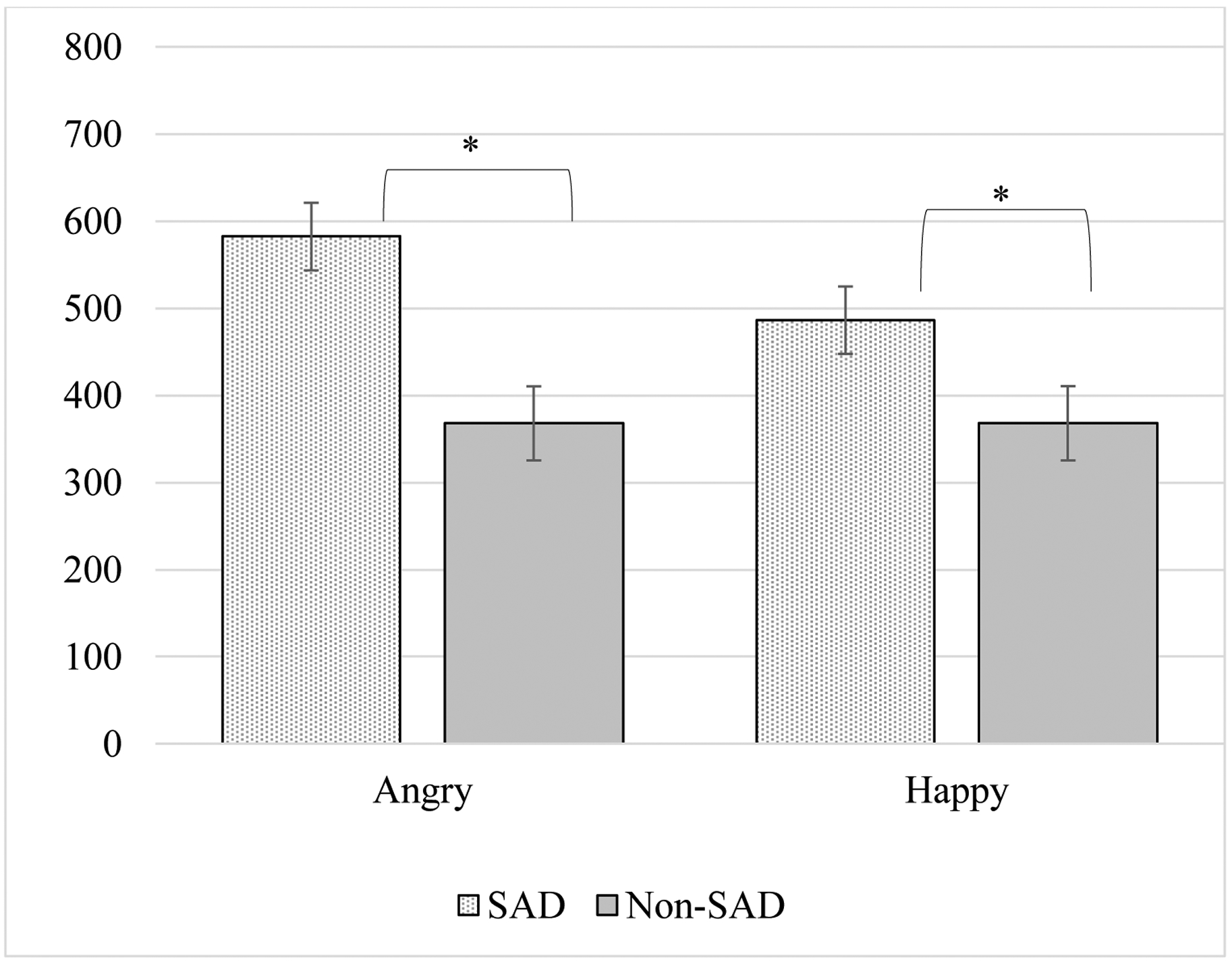
Emotion Specificity for First Fixation Duration Length to the Eye Region. Note. An asterisk indicates significant between-group difference. Error bars show standard errors.

In addition to performing independent analyses on each emotion-neutral pair separately, we also performed a repeated measures ANOVA with emotion (angry and happy eye regions) x group (SAD and non-SAD) for each metric, consistent with Shechner and colleagues (2013). Using this approach, there was a statistically significant interaction for first fixation duration, *F*(1,46) = 5.99, *p* = .018, partial eta squared = .115 (Figure 2). Specifically, duration of first fixation towards the eye region of angry faces relative to happy faces was statistically longer for adolescents with SAD versus adolescents without SAD. There were also significant main effects for both emotion type, *F*(1,46) = 5.94, *p* = .019, partial eta squared = .114 as well as group, *F*(1,46) = 9.32, *p* = .004, partial eta squared = .168. Specifically, both groups demonstrated greater first fixation duration to the eye region of angry versus happy faces, and adolescents with SAD were quicker to maintain gaze to the initially fixated eye region, irrespective of emotion, compared to adolescents without SAD. No other significant interactions were noted for the other variables of interest (i.e., total fixation duration, fixation duration across the six time epochs, and latency).

## Discussion

Our study is the first, to our knowledge, to examine visual attention to the eye region in adolescents with and without SAD. Such research during this developmental period is critical given SAD, which is characterized by deficits in social information processing, most often onsets during adolescence. We first determined that youth across groups first fixated significantly more often on the eye region compared to the mouth region, although both regions have been found to be important face regions for decoding of emotions (Eisenbarth & Alpers, 2011). For the early eye-tracking metrics (i.e., first 500ms), our results suggest that any effects observed in the current study may be unique for gaze to the eye region and not simply representative of patterns of attention to the mouth region. Across the stimulus presentation, youth across the groups spent more time looking at the eye region of angry faces relative to the mouth region of angry faces, signaling that the eye region of threat faces is attention grabbing and prioritized visually among all adolescents, irrespective of social anxiety. No differences were observed, however, for fixation duration to the eye region of happy faces versus fixation duration to the mouth region of happy faces which suggests the mouth likely serves an important function in emotion decoding for happy facial expressions (Eisenbarth & Alpers, 2011). Although there was some evidence for emotional specifiity in terms of fixation duration (i.e., overall greater mean dwell time to the eye region, but fixation duration to the mouth region was pronounced for happy faces), adolescents in our study generally spent greater time fixating on the eye region compared to the mouth region. In sum, we believe these findings offers support for our focus on the eye region specifically.

Contrary to our hypothesis that youth with SAD would exhibit more avoidance (as has been observed most often among both adults and children with SAD), we found that adolescents with SAD consistently exhibited a pattern of vigilance, marked by faster orienting towards the eye region during initial stimulus presentation relative to adolescents without SAD. Our findings are in contrast to those of Moukheiber and colleagues (2010) and Keil and colleagues (2018) who demonstrated that social anxiety was associated with avoidance of the eye region. Relative to those without SAD, adolescents with SAD demonstrated a general pattern of vigilance towards the eye region, irrespective of stimulus emotion. Although adolescents with SAD were quicker to orient to the eye region (i.e., latency metric), they did not demonstrate a greater tendency to first orient their attention to the eye region of emotional faces relative to the adolescents without SAD. At the same time, however, adolescents with SAD demonstrated greater sustained initial attention towards the eye region of angry faces (when compared with happy faces) which is indicative of ‘threat vigilance’.

Extant research suggests individuals with SAD scan the environment for signs of threat related to potential negative evaluation and detect these signs rapidly (Bögels & Mansell, 2004; Rapee & Heimberg, 1997), suggesting hypervigilant attention. Per evolutionary models, when threat is perceived as imminent, humans orient gaze to the eye region of the face in order to detect the threat source (Tipples, 2006). Hypervigilant attention towards the eye region did not differ as a function of stimulus emotion. The lack of significant differences as a function of stimulus emotion could be attributed to the eye regions of both neutral (Cooney et al., 2006; Dodd et al., 2015) and happy faces (Wieser et al., 2009) being appraised as threatening for adolescents with SAD relative to those without SAD. This lack of emotion specificity in visual attention has also been reported in other studies with adults (Staugaard, 2010) and children with SAD (Keil et al., 2018).

Results suggest that group-level differences in maintenance of initial fixations are present, regardless of emotion valence (i.e., global perseveration). For adolescents with SAD, attending to the eye region and maintaining attention there is not specific to socially threatening faces. As indicated previously by Shechner and colleagues (2013), the paired stimulus approach precludes isolation of aberrant attentional processes to one specific type of stimulus (e.g., emotional) without considering the relative influence of the concurrently presented stimulus (e.g., neutral). As such, we also examined emotion specificity. Results suggested that maintenance of the first fixation towards the eye region of angry faces relative to happy faces was statistically longer for adolescents with SAD compared to adolescents without SAD, offering some support for biases in maintenance of attention not being generalized to social stimuli but rather unique to socially threatening stimuli (e.g., eye region of angry faces).

Collectively, these findings suggest that adolescents with SAD relative to non-SAD adolescents demonstrated some differences in attention allocation to the eye region at the onset of stimulus exposure but this was not evident across stimulus presentation (i.e., no evidence for group-level differences for cumulative fixation duration nor across the six time epochs). Consistent with findings reported by others (Schmidtendorf et al. 2018; Seefeldt et al. 2014; Shechner et al. 2013), adolescents with SAD did not show systematic avoidance or maintenance of attention, compared to their non-SAD counterparts, in the later portions of the stimulus presentation. Although contrary to our hypothesis, adolescents across groups demonstrated a general tendency to prioritize attention to the eye region – largely irrespective of emotional valence, in line with the increasing salience of emotional stimuli during adolescence (Rapee et al., 2019). Differences in gaze patterns during early stimulus exposure have been found elsewhere within generally anxious youth as well as youth with SAD but lack specific focus on internal features, such as the eye region (e.g., Capriola-Hall et al., 2019; Shechner et al., 2013). In a potentially threatening context, adolescents with SAD may demonstrate atypical involuntary attentional processes (Shechner et al., 2013) and sustained vigilance over time in response to the initially fixated eye region which has not been observed in past research among children with SAD (Keil et al., 2018). These differences might be attributed to the unique developmental period sampled (e.g., adolescence) as a recent meta-analysis demonstrated that differences in attentional processing among anxious and non-anxious youth were more pronounced in adolescents than in children (Dudeney et al., 2015).

This study is not without limitations, which future research in this area should seek to address. Specifically, our sample size was relatively small. Given our moderate to large effects, failure to detect hypothesized associations could reflect Type II errors. Given the paired nature of our stimuli which only included emotional faces (happy and angry) paired with neutral faces, our analyses preclude isolation of attention biases to emotional stimuli without considering the relative influence of the concurrently presented neutral stimulus (Shechner et al., 2013). Although we have included emotional specificity analyses to help address this issue, emotional stimuli face pairs (e.g., happy paired with angry) were not presented concurrently in our study. In addition, the reliability estimates ranged from the lower limits of acceptability to the good range. The lower reliability estimates for the epoch-level analysis, in particular, might have affected our results. We note, however, that this might a broader limitation which is not unique to this current study as the internal consistencies were generally comparable to those reported by Schmidtendorf et al. (2018) and Keil et al. (2018). Another noteworthy limitation was the lack of a clinical interview or a test of cognitive ability for the non-SAD group. However, all youth in the non-SAD group completed the SCARED and were below clinical threshold on the social anxiety subscales as well as on reported total anxiety level which suggests that they were unlikely to meet diagnostic criteria for an anxiety disorder. In addition, mean scores for the anxiety subscales as well as on reported total anxiety level for the non-SAD group were comparable to mean level scores reported in past studies of healthy comparison samples (Rappaport et al., 2017). However, other mental health concerns cannot be excluded given we did not include measures of general psychopathology. Specifically, past eye-tracking studies have determined that co-occurring depression diagnoses (Gotlib, 1982) and depressive symptoms (as rated dimensionally; Keil et al., 2018) can affect face perception, specifically attention to the eyes. Although we did have diagnostic data available for our SAD group, we are unable to determine whether co-occurring depression could have potentially affected our findings given we did not formally assess for depression among our non-SAD group outside of the use of the telephone screener. Participants from both groups were predominantly Caucasian. Future studies should examine whether these findings are observed in more ethnically and culturally diverse samples. Lastly, our study relied on a free viewing eye-tracking paradigm which limits our study’s ecological validity relative to real world social interaction. As such, future research should use more innovative ecologically valid eye-tracking approaches (e.g., mobile eye-tracking during in-vivo situations; Allen et al., 2019).

Notwithstanding these limitations, our results suggest patterns of social information processing (i.e., vigilance towards eye region during early stimulus exposure) that may be specific to adolescents with SAD. Although avoidance of social evaluation cues has traditionally been regarded as a hallmark of SAD, our results suggest that heightened attention, perhaps vigilance, characterizes SAD in adolescence. There was some evidence for general attending towards the eye region irrespective of stimulus valence for youth with SAD compared to youth without SAD. However, the teens with SAD had longer initial fixation duration (i.e., lack of visual disengagement) to the eye region of angry faces, relative to happy faces. In conclusion, these findings suggest that the eye region represents a salient facial feature that draws special attention for adolescents with SAD, perhaps because the eyes provide a signal of potentially threatening social evaluation.

## Figures and Tables

**Table 1:** Participant Characteristics

	SAD (*n* =28)	Non-SAD (*n* =25)	*X*^2^/*t*
Gender (female)	18	15	.769
Race			.211
White	20	21	
Black	1	1	
Hispanic	2	0	
Asian/Other	0	0	
Other	5	3	
Age (years)	14.07	13.56	1.399
SCARED Social Anxiety Subscale	9.58	2.88	8.45**
SCARED Total Score	33.79	10.00	7.40**
BFNE Straightforward Total Score	26.77	14.40	5.64**

^ *p* < .10;

* *p* < .05;

** *p* < .01

Note. SAD = Social Anxiety Disorder. SCARED = Screen for Child Anxiety Related Disorders, Child Version; BFNE = Brief Fear of Negative Evaluation

**Table 2:** Cronbach’s Alpha for Eye-tracking Metrics

	Angry-Neutral Face Pairs		Happy-Neutral Face Pairs		
	Angry	Neutral	Happy	Neutral	All Stimuli
First Fixation Direction	.69		.71		.84
Latency	.73	.68	.56	.80	.91
First Fixation Duration	.88	.88	.86	.88	.97
Total Dwell Time	.93	.92	.89	.89	.94
0–500ms	.59	.45	.49	.51	.85
500–1000ms	.73	.57	.70	.38	.88
1000ms–15000ms	.62	.57	.60	.59	.87
1500ms–2000ms	.61	.65	.46	.62	.83
2000ms–2500ms	.52	.55	.55	.47	.83
2500ms–3000ms	.44	.68	.64	.40	.81

**Table 3:** Means and Standard Deviations for Attention Bias Variables by Group for Angry-Neutral Eye Region

Anger Eye Region	SAD (*n* = 25) *M* (*SD*)	Non-SAD (*n* = 25) *M* (*SD*)	*t*	*d*
Latency	413.37 (136.54)	610.36 (160.95)	4.82**	1.32
First Fixation Duration	583.11 (223.83)	367.69 (191.78)	3.74*	1.04
First Fixation Direction (Anger-Neutral)^a^	.45 (.24)	.45 (.29)	−.04	.02
Total Fixation Duration	642.13 (283.72)	635.86 (285.94)	.08	.02
0–500ms	58.11 (37.64)	69.50 (54.41)	−.85	.24
500–1000ms	136.21 (79.74)	124.80 (76.48)	.52	.15
1000ms–15000ms	113.51 (70.92)	102.04 (57.86)	.63	.18
1500ms–2000ms	118.25 (66.83)	102.70 (47.58)	.95	.27
2000ms–2500ms	97.85 (60.46)	107.07 (60.23)	−.54	.15
2500ms–3000ms	102.73 (60.42)	113.35 (64.14)	−.60	.17
Neutral Paired with Anger Eye Region				
Latency	543.85 (237.23)	697.88 (265.28)	2.23*	.61
First Fixation Duration	611.64 (290.73)	637.76 (314.85)	.31	.08
Total Fixation Duration	611.64 (290.73)	637.76 (314.85)	.31	.08
0–500ms	74.07 (49.40)	50.44 (42.37)	1.80^	.51
500–1000ms	99.41 (58.85)	90.21 (62.25)	.54	.15
1000ms–15000ms	110.08 (67.09)	105.58 (70.67)	−.85	.07
1500ms–2000ms	105.58 (70.67)	118.63 (52.79)	−.74	.21
2000ms–2500ms	115.78 (65.07)	123.41 (55.97)	−.44	.13
2500ms–3000ms	111.79 (73.34)	94.43 (56.81)	.94	.26

^ *p* < .10;

* *p* < .05;

** *p* < .01; Note. SAD = Social Anxiety Disorder

a Given that first fixation direction toward angry faces was expressed as a proportion (i.e., number of trials gaze was first directed to angry face *divided* by total number of trials with eye movements to angry-neutral face pairs) which equaled 100%, there was no need to enter first fixation direction proportion to neutral faces given that the proportion was calculated with this percentage already included (i.e., exclusion of redundant information because derived from other variable retained in the data set).

**Table 4:** Means and Standard Deviations for Attention Bias Variables by Group for Happy-Neutral Eye Region

Happy Eye Region	SAD (*n* = 25) *M* (*SD*)	Non-SAD (*n* = 25) *M* (*SD*)	*t*	*d*
Latency	440.67 (195.38)	618.47 (181.40)	3.42*	.94
First Fixation Duration	486.17 (179.06)	367.88 (198.48)	2.28*	.63
First Fixation Direction (Happy-Neutral)^a^	.46 (.26)	.54 (.32)	−.90	.25
Total Fixation Duration	509.43 (286.47)	483.01 (274.01)	.34	.09
0–500ms	50.64 (37.93)	38.11 (39.26)	1.15	.32
500–1000ms	100.03 (81.96)	82.29 (69.61)	.83	.23
1000ms–15000ms	75.98 (51.23)	81.61 (60.49)	−.36	.10
1500ms–2000ms	71.78 (47.06)	82.01 (54.13)	−.71	.20
2000ms–2500ms	87.20 (55.05)	92.97 (53.64)	−.38	.11
2500ms–3000ms	107.33 (72.08)	97.41 (53.12)	.55	.16
Neutral Paired with Happy Eye Region				
Latency	477.98 (187.95)	660.24 (222.77)	3.23*	.88
First Fixation Duration	550.38 (192.47)	362.57 (161.48)	3.83*	1.06
Total Fixation Duration	566.64 (233.26)	576.31 (236.21)	.15	.04
0–500ms	56.82 (32.33)	55.67 (47.38)	.10	.03
500–1000ms	81.82 (50.17)	90.57 (52.86)	−.60	.17
1000ms–15000ms	114.72 (52.78)	84.29 (58.88)	1.92^	.54
1500ms–2000ms	120.98 (64.73)	82.91 (56.03)	2.22*	.63
2000ms–2500ms	101.06 (51.05)	83.95 (49.29)	1.21	.34
2500ms–3000ms	81.95 (51.44)	89.85 (53.59)	−.53	.15

^ *p* < .10;

* *p* < .05;

** *p* < .01; Note. SAD = Social Anxiety Disorder

a Given that first fixation direction toward happy faces was expressed as a proportion (i.e., number of trials gaze was first directed to happy face *divided* by total number of trials with eye movements to happy-neutral face pairs) which equaled 100%, there was no need to enter first fixation direction proportion to neutral faces given that the proportion was calculated with this percentage already included (i.e., exclusion of redundant information because derived from other variable retained in the data set).

## Footnotes

1 The other metrics (latency and first fixation duration) were not examined given limited power to run analyses since repeated measures ANOVA will exclude data from subjects who only allocated attention to the eyes; specifically, latency and first fixation duration have missing data because if attention was not first allocated to the eye region, it is coded as a missing value (e.g., “999” or “NaN”) not a “0” value. A “0” value would skew latency and first fixation duration averages since those metrics rely on *time*, not *number* of trials.
